# Database Development of SiO_2_ Etching with Fluorocarbon Plasmas Diluted with Various Noble Gases of Ar, Kr, and Xe

**DOI:** 10.3390/nano12213828

**Published:** 2022-10-29

**Authors:** Youngseok Lee, Heejung Yeom, Daehan Choi, Sijun Kim, Jangjae Lee, Junghyung Kim, Hyochang Lee, ShinJae You

**Affiliations:** 1 Department of Physics, Chungnam National University, Daejeon 34134, Korea; 2 Korea Research Institute of Standards and Science, Daejeon 34113, Korea; 3 Samsung Electronics, Hwaseong-si 18448, Korea; 4 Institute of Quantum Systems (IQS), Department of Physics, Chungnam National University, Daejeon 34134, Korea

**Keywords:** plasma etching, plasma chemistry, noble gas, plasma diagnostics

## Abstract

In the semiconductor industry, fluorocarbon (FC) plasma is widely used in SiO_2_ etching, with Ar typically employed in the dilution of the FC plasma due to its cost effectiveness and accessibility. While it has been reported that plasmas with other noble gases, namely Kr and Xe, have distinct physical properties such as electron density and temperature, their implementation into plasma etching has not been sufficiently studied. In this work, we conducted SiO_2_ etching with FC plasmas diluted with different noble gases, i.e., FC precursors of C_4_F_8_ and CH_2_F_2_ with Ar, Kr, or Xe, under various gas flow rates of each as well as plasma diagnostics for the process interpretation. We show that Ar, Kr, and Xe gas mixtures depend on the FC precursor flow rate and the pattern width in a significantly different manner and we elucidate these findings based on plasma diagnostic results. The results of this work are expected to offer a practical etching database for diverse applications including plasma process engineering and the development of plasma simulation in the semiconductor industry.

## 1. Introduction

Plasma is of increasing importance in semiconductor manufacturing that proceeds through multiple and repetitive processes such as lithography, deposition, and etching [1]. In particular, as the feature size of transistors continues to shrink to meet market demands for higher device performance, the etch process should achieve critical dimensions on the sub-ten nanometer scale for next-generation semiconductors, leading plasmas to play a bigger role in the industry [2].

The etching of SiO_2_, which is one of the most widely used materials in semiconductor devices, has been intensely studied for decades in terms of etch rate, selectivity, etc. In plasma SiO_2_ etching, halogen-containing precursors are employed to generate volatile etch products such as silicon halide. Gas mixtures with Br and Cl have been studied for SiO_2_ etching to overcome the disadvantage of isotropic etching from the use of F-containing precursors such as SF_6_ [3]. Perfluorocarbon (PFC) gases such as CF_4_ and C_4_F_8_ are widely used owing to their characteristic behaviors that improve the etch selectivity of SiO_2_ over other materials including Si and Si_3_N_4_, as well as their achievement of etch anisotropy by forming fluorocarbon (FC) films on the surface of SiO_2_ trench sidewalls [4]. Although there have been numerous reports on the development of alternative precursors to the conventional PFC gases due to their high global warming potential [5,6,7,8], the conventional PFC gases such as C_4_F_8_ still remain indispensable in plasma SiO_2_ etching for denser patterning.

The addition of H_2_ or O_2_ gas in PFC plasmas has been widely employed to control the etching characteristics; while H atoms generated through the dissociation of H_2_ in plasma act as a scavenger for F atoms, thereby lowering etch rates and increasing SiO_2_-to-Si selectivity, O atoms from O_2_ dissociation consume C atoms on the material surfaces, reducing the formation of FC films [9]. Following the same mechanism as H_2_/O_2_ addition, hydrofluorocarbon gases (HFC, CH_x_F_4−x_ with x = 1, 2, or 3) with H atoms in their molecular structures instead of F atoms are frequently adopted, and numerous studies on the mixture of PFC and HFC precursors have been reported over the years [10,11,12,13,14,15,16,17,18].

Fluorocarbon precursors, referring to one or both of the PFC and HFC precursors in this paper, are typically diluted with noble gases when used in SiO_2_ etching to control the extent of the polymeric properties of the plasma. While Ar is the most widely used for FC precursor dilution due to its low cost and accessibility, other noble gases have been examined as diluting gases following reports that plasmas of various noble gases have significantly different characteristics as compared with Ar plasmas, which mainly originate from differences in mass and ionization energy [10,19,20]. The employment of diverse noble gas species in FC precursor dilution can, thus, be another process condition control knob to adjust the physical and chemical properties of the plasma via variation of plasma parameters including electron density and temperature. In particular, among the various noble gas species, Kr and Xe are attracting more research interest since their use in etching SiO_2_ with FC plasmas has been relatively less reported as compared with the numerous studies on their physical and chemical properties [19,20,21]. Therefore, there is a need to develop a plasma etch database with respect to various gas mixtures of different noble gases including Kr and Xe.

In this work, we present experimental results of plasma SiO_2_ etching with various gas mixtures of C_4_F_8_, CH_2_F_2_, and O_2_ diluted with Ar, Kr, and Xe to provide a SiO_2_ etching database, which has been relatively little studied. Changes in the plasma parameters from process condition variations are identified via various plasma diagnostic methods to interpret the resulting etch profiles. The scanning electron microscope (SEM) results show significant changes in the etch profiles according to the process condition variations with respect to the noble gases.

## 2. Experiments

### 2.1. Description of the Processing Chamber

**Figure 1:**
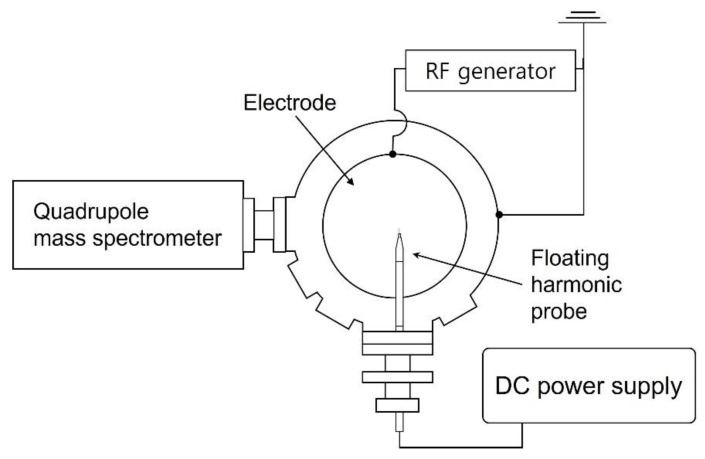
Top schematic view of the plasma etching chamber and plasma diagnostic tools.

Figure 1 illustrates a schematic of the plasma etching chamber. For etching, coupon wafers with a diameter of 300 mm are loaded on the electrode and 13.56 MHz radiofrequency (RF) power of 500 W is applied. A turbomolecular pump evacuates the chamber, resulting in a base pressure of approximately 10^−5^ Torr before etching, and a throttle valve maintains the processing pressure at 28 mTorr with gases injected by mass flow controllers through a showerhead that faces the electrode and acts as the grounded electrode (not shown in Figure 1). Further details are described in our previous reports [21,22,23]. The reference gas mixture examined in this work consists of C_4_F_8_, CH_2_F_2_, O_2_, and Ar with flow rates of 6, 6, 3, and 60 sccm, respectively. An increase in the gas flow rate of either the noble gas (Ar, Kr, or Xe) or the FC precursors is adopted in this work for process condition variation. As also shown in Figure 1, the plasma diagnostic tools, namely a floating harmonic probe and quadrupole mass spectrometer (QMS) (PSM, Hidden Analytical, Warrington, UK), are mounted after plasma processing.

### 2.2. Sample Preparation

**Figure 2:**
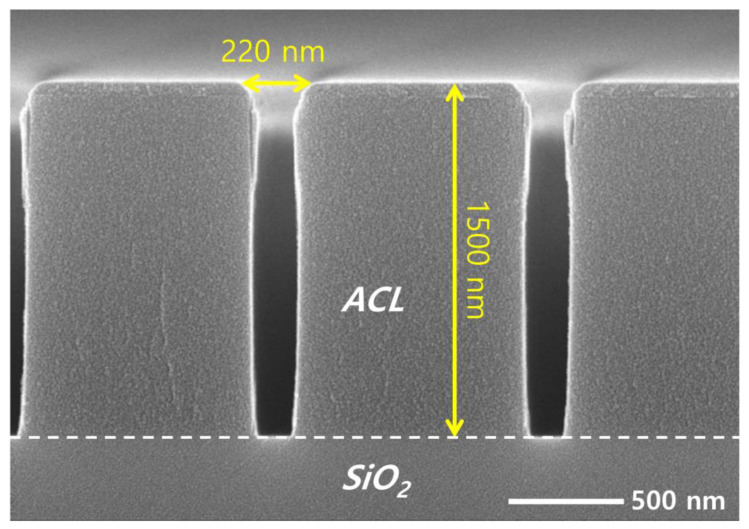
SEM image of the mask pattern on the target silicon dioxide before etching.

The coupon wafers are prepared for etching by dicing patterned wafers with a diameter of 300 mm into 1 × 2 cm^2^ pieces. An amorphous carbon layer (ACL) of 1500 nm deposited on SiO_2_ acts as an etch mask, having line patterns with different widths of 185, 196, 221, and 240 nm. Figure 2 shows a cross-sectional view of a coupon wafer with a pattern width of 220 nm, obtained using a SEM. While most SEM images of the etch results shown in this article are the 220 nm width, we briefly address the effect of the pattern width on the etch profile in Section 3.

### 2.3. Plasma Diagnostic Methods

#### 2.3.1. Electron Density Measurement

We employ a floating harmonic probe for the electron density measurements, which has been reported to be suitable for processing plasma diagnostics where the deposition of polymer films deteriorates the performance of other plasma diagnostic tools such as the Langmuir probe [24]. The working principle of the floating harmonic probe is briefly described below.

Plasma current collected in the biased metallic tip of the probe can be expressed as modified Bessel functions that represent the harmonic components of the current originating from the oscillation of the plasma potential [25]. Blocking the direct current (DC) component of the current, i.e., $i_{DC}=i^{+}-i^{-}\exp\left[e\left(\bar{V}-V_{p}\right)/T_{e}\right]I_{0}\left(eV_{0}/T_{e}\right)=0$, the plasma current $i_{pr}$ has the form $i_{pr}=-2i^{-}\exp\left[e(\bar{V}-V_{p}\right)T_{e}]\left(i_{1\omega }+i_{2\omega }+\cdots \right)$, where $i^{-}$ is the ion saturation current, $\bar{V}$ is the DC bias voltage, $T_{e}$ is the electron temperature, ω is the voltage frequency applied to the probe, and $i_{n\omega }$ is the *n*-th harmonic components of the current [24]. Thus, the ion density can be calculated using the following equation:

(1) $$i_{1\omega }=-2i^{+}\left(I_{1}/I_{0}\right)\cos\left(\omega t\right)=-2\left(0.61en_{i}u_{B}A\right)\left(I_{1}/I_{0}\right)\cos\left(\omega t\right),$$

where $n_{i}$ is the ion density, $u_{B}$ is the Bohm velocity, and *A* is the probe area. The first harmonic current measurement provides the ion density with Equation (1).

#### 2.3.2. Radical and Ion Density Measurement

FC radical densities such as CF_2_ and CF_3_ are measured with a QMS (PSM, Hiden Analytical, Warrington, UK), one of the most widely used plasma diagnostic tools. Gas-phase neutral species should be ionized before entering the mass filter, which is a quadrupole with two pairs of electrodes biased with opposite RF and DC voltages. The applied RF and DC voltages determine which mass will pass the quadrupole filter [26]. Details of the working principle can be found in numerous reports [22,27,28,29]. The QMS chamber is separated from the main chamber via an orifice with a diameter of 150 µm, and the QMS chamber pressure in the present work is maintained under 10^−6^ Torr during the measurements with a differential pumping unit. While the filament in the QMS ionizer emits thermionic electrons for the ionization of radicals, it is not necessary for the plasma ion measurements. Instead, an energy filter, normally termed a Bessel box, is used to obtain the ion energy distribution for specific ions with a target mass. Note that the measured radical and ion signals are quantified by normalizing them to the gas density calculated by the ideal gas law and the electron density obtained by the floating harmonic probe, respectively [22].

## 3. Results and Discussion

### 3.1. Effects of Noble Gas Species

**Figure 3:**
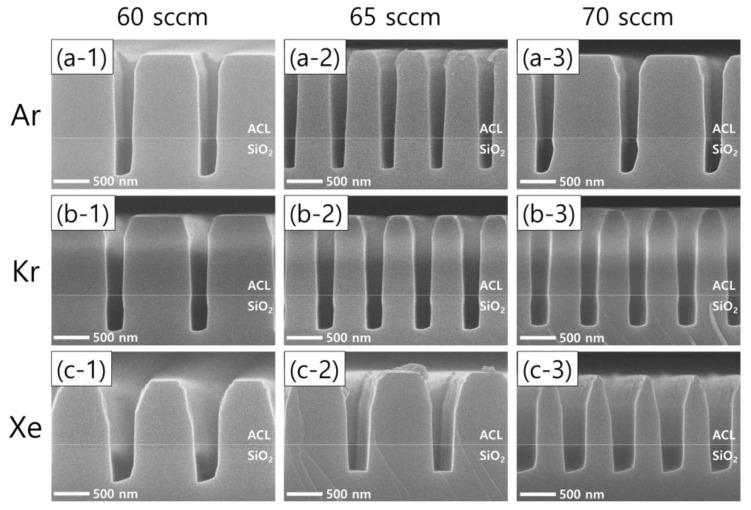
SEM images of the etch profile evolution with different noble gas species of (**a**) Ar, (**b**) Kr, and (**c**) Xe at varying flow rates of 60 sccm (**left column**), 65 sccm (**middle column**), and 70 sccm (**right column**). The flow rates of the FC precursors and O_2_ are 6 sccm and 3 sccm, respectively.

Figure 3 shows the results of SiO_2_ etching with the addition of different noble gas species at different flow rates. The left, middle, and right columns represent noble gas flow rates of 60, 65, 70 sccm, respectively, while the top, middle, and bottom rows represent the addition of Ar, Kr, and Xe, respectively. Leaving the asymmetric SiO_2_ etch profiles observed with Ar and Xe addition aside, it is worth noting that the remaining ACL mask profiles are found to depend on the noble gas species. The use of Xe instead of Ar or Kr leads to a wider mask pattern width, which means less selective etching.

**Figure 4:**
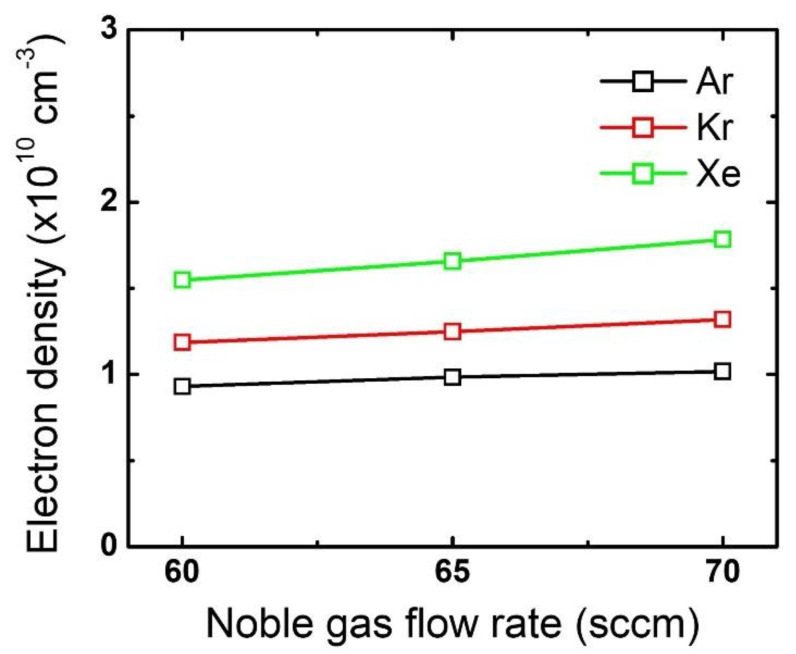
Electron density obtained from different noble gas species by flow rate. The flow rates of the FC precursors and O_2_ are 6 sccm and 3 sccm, respectively.

The difference in the mask etch profiles according to the noble gas variation can be explained by using the plasma diagnostic results. Figure 4 plots the changes in the electron density by noble gas species and flow rate variation. While the electron density barely changes with an increase in the noble gas flow rate, the different noble gases themselves show significant differences: electron density is highest for Xe, then Kr, and then Ar. This trend stems from the different ionization threshold energies among the noble gases [19,20,30]. Since a higher electron density reflects that more positive ions bombard the substrate, it is considered that the mask experiences the most intense ion bombardment when Xe is added to the gas mixture. Moreover, assuming all ions obtain the same kinetic energy while being accelerated in the sheath, Xe ions having the highest mass among the tested noble gases would provide the highest momentum transfer to the mask.

**Figure 5:**
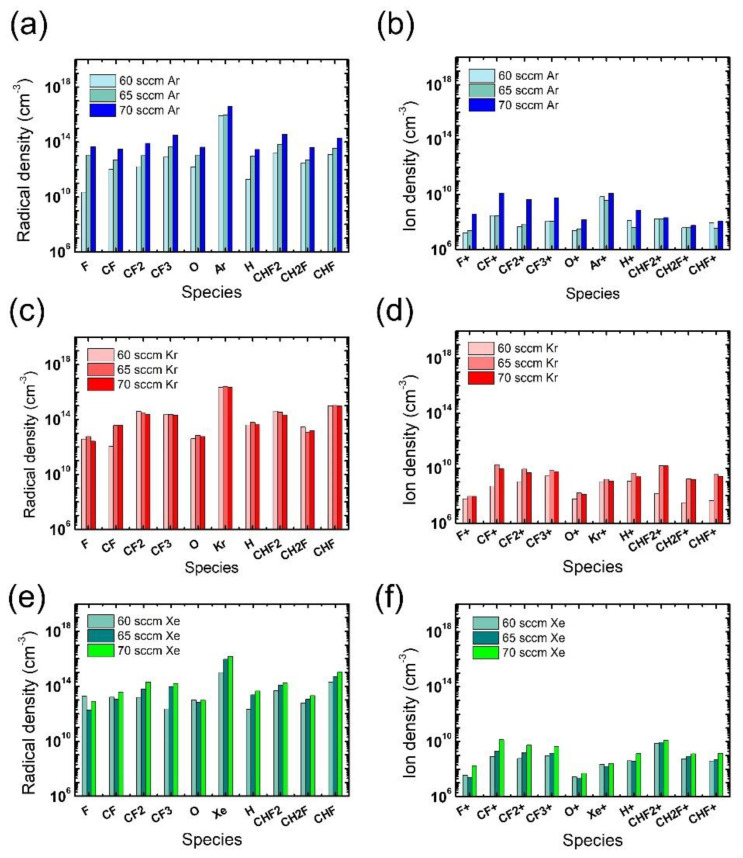
Radical (**left**) and ion (**right**) densities obtained from (**a**,**b**) Ar-mixture plasma, (**c**,**d**) Kr-mixture plasma, and (**e**,**f**) Xe-mixture plasma, at various noble gas flow rates. The flow rates of the FC precursors and O_2_ are 6 sccm and 3 sccm, respectively.

In addition to the electron density, radical and ion densities with different noble gases at different flow rates were measured, as shown in Figure 5. Among the various chemical species in Figure 5a,b, the O radicals are expected to have the greatest impact on the ACL mask etching [31], yet no notable difference is found in the measured O radical density with different noble gases at different flow rates. Thus, we consider that the widest mask pattern width observed in the etch results with the Xe addition might be attributed to the more intense ion bombardment from Xe than from Ar or Kr, which enhances the chemical reactions between O radicals and the mask. The discussion about the effects of noble gas species is summarized in Table 1.

### 3.2. Effects of FC Precursor Flow Rate

**Figure 6:**
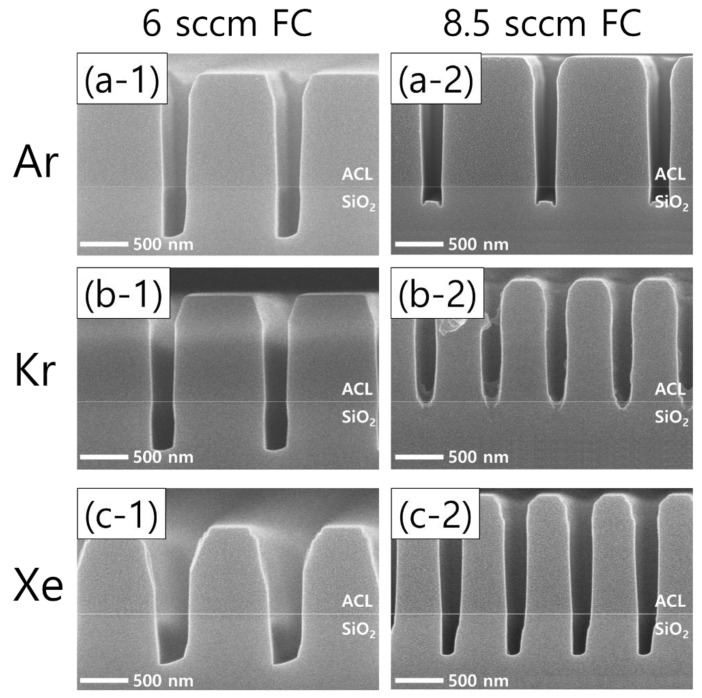
SEM images of the etch profiles with different noble gas species of (**a**) Ar, (**b**) Kr, and (**c**) Xe, at a fixed flow rate of 60 sccm. The flow rate of the FC precursors is 6 sccm (**left column**) and 8.5 sccm (**right column**), while the O_2_ flow rate is 3 sccm.

An increase in the flow rate of the FC precursors (both C_4_F_8_ and CH_2_F_2_) exhibits interesting etch profile evolutions. Figure 6 shows the etch results from an increased FC precursor flow rate from 6 sccm (left column) to 8.5 sccm (right column) with Ar, Kr, and Xe of 60 sccm (top, middle, and bottom rows, respectively). It can readily be seen that increasing the FC flow rate results in significantly different etch profiles among the cases of different noble gases.

Looking at the Ar results, microtrenching is found as the FC flow rate increases, which is considered to derive from more ions colliding with the trench sidewalls that have shrunk by increased FC film deposition, allowing the etch rate at the corners of the trench bottoms to increase [32]. The effects of the FC flow rate increase are more pronounced in the cases of Kr and Xe than Ar. With Kr, the FC flow rate increase immediately results in etch stops of the trench bottoms. In a similar but less effective way, the etch profile evolution with Xe shows narrower pattern widths and SiO_2_ etch profiles. Comparing the changes in the Kr and Xe cases with those of the Ar case, it is found that Kr and Xe plasmas are more sensitive to changes in the flow rate of the FC precursor gas mixtures. The etch profile evolutions shown in Figure 6 will be briefly discussed with the plasma diagnostics results.

**Figure 7:**
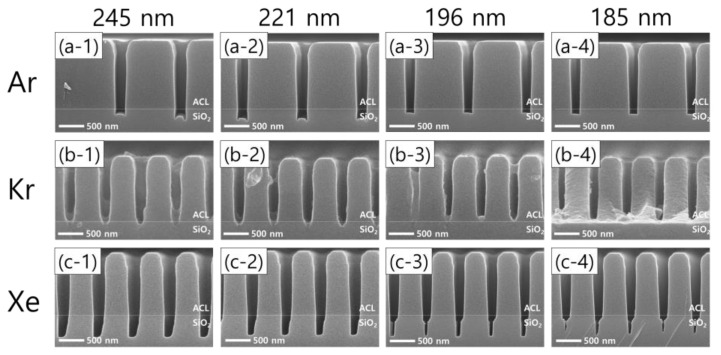
SEM images of the etch profile evolution with different noble gas mixtures of (**a-1**–**a-4**) Ar, (**b-1**–**b-4**) Kr, and (**c-1**–**c-4**) Xe, by pattern width.

Moreover, significant differences in the etch profiles among noble gas species are observed as the pattern width shrinks with the FC flow rate of 8.5 sccm, as shown in Figure 7. Taking the Ar cases first, Figure 7(a-1–a-4) show that microtrenching disappears with narrowing pattern widths. In other words, an increase in the pattern width has the same effect as an increase in the FC precursor flow rate, which reflects that the wider pattern widths are, the higher the FC radical fluxes are in the trenches. Meanwhile, the etch stop with Kr, shown in Figure 6(b-2), is also found with different pattern widths, as shown in Figure 7b. As for Xe, one interesting profile evolution behavior is observed. With decreasing pattern widths from 245 nm to 185 nm, the trench bottoms become narrower, leaving seam-like holes near the center of the trench bottoms. This feature has not been reported to the best of our knowledge. The seam-like etch profile is not clearly understood here, requiring more rigorous investigations in the future.

**Figure 8:**
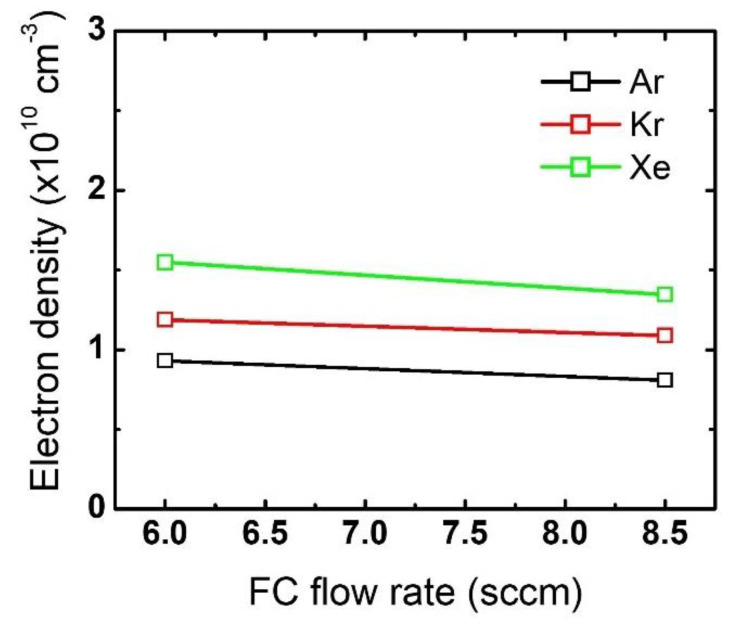
Electron density obtained from different FC gas flow rates. The flow rates of the noble gases and O_2_ are 60 sccm and 3 sccm, respectively.

Changes in electron density with an increase in the FC flow rate are plotted in Figure 8. Note that the electron density at the 6 sccm FC flow rate is the same as the one shown in Figure 4. As the FC flow rate increases from 6 sccm to 8.5 sccm, the electron density slightly decreases for all the cases with different noble gas mixtures. These changes in electron density with FC flow rate increase explain the evolution of the etch profiles in Figure 6, which was not observed with the noble gas flow rate variations.

**Figure 9:**
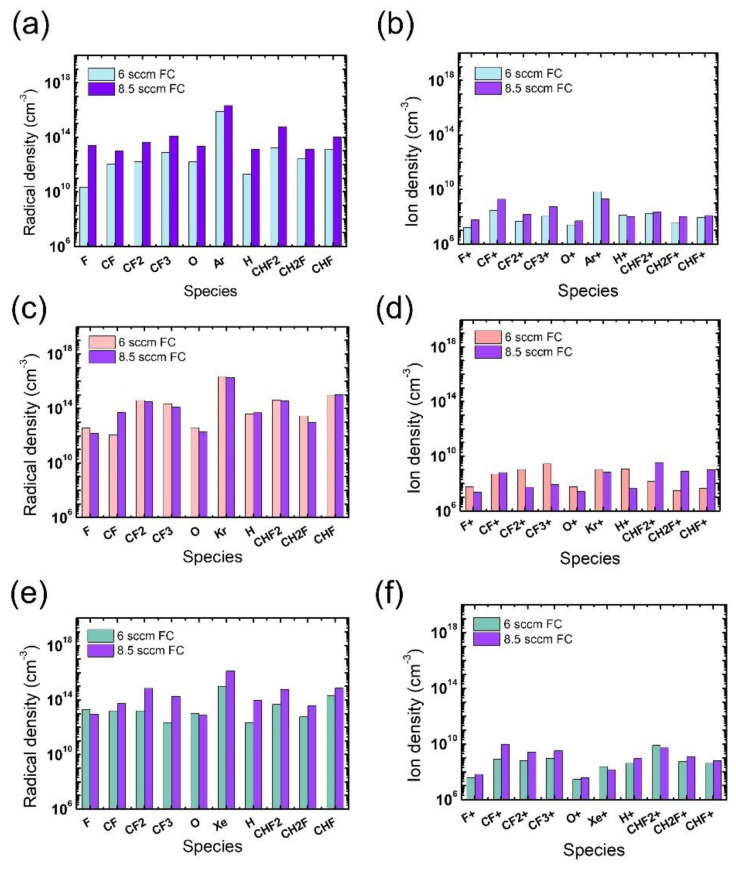
Radical (**left**) and ion (**right**) densities obtained from (**a**,**b**) Ar-mixture plasma, (**c**,**d**) Kr-mixture plasma, and (**e**,**f**) Xe-mixture plasma, at various FC precursor flow rates. The O_2_ flow rate is 3 sccm.

The radical and ion densities of the gas mixtures with an FC flow rate of 8.5 sccm were measured and compared to those for the 6 sccm FC flow rate in Figure 9. It is seen in Figure 9a,b that the FC flow rate increase with the Ar mixture leads to overall increases in both radical and ion density, driving an evolution of the microtrenching profiles shown in Figure 6(a-2). For the Kr mixture, changes in the ion density with FC flow rate increase are more notable than those in the radical density. With barely changing radical densities, the density of PFC ions significantly decreases while that of HFC ions increases, as shown in Figure 9c,d, respectively. The etch condition transition shown in Figure 6(b-1,b-2) with the increasing FC flow rate might be elucidated with this notable trend of varying ion densities; the increase in the H-containing ions, namely CHF_2_^+^, CH_2_F^+^, and CHF^+^, can significantly impede SiO_2_ etching since they are more polymeric than non H-containing FC ions [17], and this phenomenon could be significant in trench bottoms due to their directionality. For the gas mixture with Xe, the changes in the radical density are more remarkable than those in ion density. The increase in FC radical densities can be more effective in the mask region than near the etch bottom of the trench, as shown in Figure 6(c-2), where mask etching is reduced possibly due to an enhanced passivation of the mask sidewall by the increase in FC radical densities. The discussion about the effects of FC precursor flow rates and pattern widths is summarized in Table 2.

## 4. Conclusions

We examined FC plasma SiO_2_ etching with gas mixtures of FC precursors (C_4_F_8_ and CH_2_F_2_), O_2_, and a noble gas (Ar, Kr, or Xe) under different gas flow rates of either the FC precursors or the noble gas. This work was conducted to address the lack of an etch database for the additions of Kr and Xe to gas mixtures in SiO_2_ etching, despite the fact that the addition of Kr and Xe in place of Ar has been reported to have promising potential as another process control knob. In the present results, replacing Ar with Kr or Xe is found to lead to significant differences in the etch profiles, especially when the etch environment is highly polymeric. These results reflect that changes in the plasma parameters according to the noble gas species provide more opportunities to vary the plasma processing conditions, which were identified via various plasma diagnostic methods in this work.

The remarkable evolution of the etch profiles observed with decreasing pattern widths in the Xe-added polymeric plasma is worth noting again. As the pattern width shrinks, the SiO_2_ is only etched near the center of the trench bottoms, eventually leaving seam-like, narrow etch profiles. This interesting etch profile is not observed with Ar or Kr. Thus, Xe is expected to be the key factor for the seam-like etch profile, but the underlying mechanism is currently out of scope. Theoretical and experimental approaches to finding the mechanism will be explored in future work.

## Figures and Tables

**Table 1:** Summary on the discussion about the effects of noble gas species.

Gas Species	Atomic Mass	Ionization Threshold Energy	Electron Density	Momentum Transfer Rate	Mask Opening
Ar→Kr→Xe	Increase	Decrease	Increase	Increase	Increase

**Table 2:** Summary on the discussion about the effects of FC precursor flow rates and pattern widths.

Gas Species	FC Flow Rate	Radical Density	Ion Density	Trench Profile	Pattern Width	Trench Profile
Ar	Increase	Increase	Increase	Microtrenching		Microtrenching disappeared
Kr		Barely change	PFC—decrease HFC—increase	Etch stop	Decrease	Etch stop
Xe		Increase	Barely change	Narrowing		Seam-like etching

## Data Availability

The data presented in this study are available on request from the corresponding author.
